# Editorial

**DOI:** 10.1080/17513758.2013.853554

**Published:** 2013-11-18

**Authors:** Fabio A. Milner

**Affiliations:** Arizona State University, Tempe, AZ, USA

This volume contains seven articles authored or co-authored by participants in the meeting Mathematical Innovative Methods and Models Of BIOsciences (MIMMO-BIO) held in Trento, Italy, 19–21 December 2011. The unusual – and perhaps not entirely grammatically correct – name of the meeting was created to suggest, not very subtly, that it was meant to be a celebration of MIMMO Iannelli's life and professional work in analysis and mathematical biosciences for more than 40 years.

The scientific contributions of Mimmo Iannelli in many areas of analysis and mathematical biology have impacted many students who obtained their doctorates under his guidance, and many others – students and researchers at all levels in their careers – who grew in their professional development though interactions and collaborations with him. Mimmo was one of the pioneers of mathematical biology in Italy and led many collaborative research efforts locally, nationally, and internationally, both within the European Union and outside. He has had, and continues to have, collaborators and co-authors from many different countries, and is a frequent presence in national and international conferences on applied and computational mathematics, biometrics, ecology and environment, mathematical biology, and population dynamics. Mimmo is widely recognized as an accomplished mathematician, but he is also an artist, political satirist, a poet at heart, and a great cook, and has a deep knowledge and appreciation of literature, history, philosophy and politics that has kept us – his long-time friends – engaged in conversations on very diverse topics well into the night on many occasions.

The work represented here includes two articles dealing with the stability of steady-states for models of populations or ecological systems – one analysing a numerical approach to equilibrium analysis for Volterra functional equations coupled with delay differential equations and the other providing insights on how to maximize the information provided by linear stability analysis in Hopf bifurcations; two articles are concerned with ecological systems – one with the numerical simulation of the spread of rabies in raccoons and the other one with competitive exclusion in a vector–host epidemic model with a distributed delay; another two articles are devoted to different aspects of cellular behaviour – one dealing with cell-to-cell adhesion in higher dimensional space and the other one with cross-diffusive motion of bacteria following a chemoattractant signal in a non-homogeneous stratified medium; the final paper studies dynamical properties of maps fitted to time-series data in the noise-free limit. They comprise a very small sample of the numerous fields in which Mimmo himself has worked and published.

Breda *et al.* extended an idea developed earlier by his group in Udine for the analysis of the stability of equilibrium points of systems of differential and integral equations. Instead of establishing the asymptotic stability by finding the roots of the characteristic equation of the linearization around the equilibrium, they used a pseudo-spectral method to approximate the eigenvalues of the infinitesimal generator of the continuous semigroup formulation for the linearized problem. It is known that when all eigenvalues of the infinitesimal generator lie on the left half of the complex plane the equilibrium is asymptotically stable; conversely, when one of them has positive real part the equilibrium is unstable. This almost-equivalence between stability and the sign of the real part of eigenvalues was applied in the new paper to reliably approximate a bifurcation diagram for models including delay differential equations. The authors suggested that the method is also suitable for a much larger class of models, including neutral- and mixed-type equations.

Diekmann and Korvasová provided in their article evidence of the advantage of working with two free parameters rather than one in studies of Hopf bifurcations in order to maximize the information obtained. They also showed the advantage of first considering coefficients in the characteristic equation as parameters and then attempting to invert the map that defines the coefficients in terms of the parameters as they appear in the original equation. Even though their approach does not provide any information about periodic solutions away from the bifurcation point, it does yield exact information for the linearized model about stability changes of equilibrium points.

Gerardo-Giorda *et al.* applied an susceptible-exposed-infectious-recovered (SEIR) epidemic model with heterogeneous spatial diffusion to describe population mobility in a realistic landscape. The authors described how to model different landscape regions and used a mixed implicit/explicit finite-element method (with stiffness treated implicitly and the nonlinearity treated explicitly) to model the raccoon rabies epidemics in the state of New York. They presented a simulation of the epidemic from 1990 to 1994, showing very good agreement with real-life data.

Martcheva *et al.* analysed a multi-strain model for a vector-borne epidemic with a distributed delay. They showed that the system always admits a disease-free equilibrium (DFE), and that a dominant-strain equilibrium exists when the basic reproduction number of the strain with the largest one (i.e. *dominant*) is greater than unity. The authors defined a reproduction number for each of the strains and then called *reproduction number of the epidemic* the largest of them. They proved that when the latter is less than unity, the DFE is globally stable and, when it is greater than unity it is unstable. In that case, the dominant-strain equilibrium is locally asymptotically stable. Finally, the authors proved competitive exclusion under the stronger assumption that both the host-to-vector and the vector-to-host reproduction numbers of the dominant strain are maximal among all strains.

Dyson *et al.* contributed a partial differential equation (PDE) model for cell-to-cell adhesion with a nonlocal flux term that models the component of cell motion attributable to cells having formed bonds with other nearby cells. The authors proved local existence of solutions using the theory of fractional powers of analytic semigroup generators. They then established the positivity and boundedness of solutions and used these to prove global existence. Finally, they studied the asymptotic behaviour of solutions about the spatially uniform state and they illustrated the applicability of the model by simulations of *in vitro* wound closure experiments.

Marinoschi considered a model of the cross-diffusion motion in a population of bacteria driven by a chemoattractant signal in a non-homogeneous stratified medium with *n* layers. The model consists of *n* systems of nonlinear parabolic PDEs with transmission boundary conditions between layers. She proved the well-posedness of anasymptotic solution with respectto the small parameter of the problem and established the existence of a global-in-time solution for arbitrarily large initial data. Finally, she considered a control problem of reducing the chemoattractant concentration below a threshold by controlling the initial distribution of the bacteria. She then proved the existence of a solution to the control problem and determined the optimality conditions.

Lindström considered a problem of model selection for time-series analysis. He addressed the fundamental questions of existence of amethod for classifying the long-term qualitative behaviour from finite time-series data and, if one does exist, what amount of data and what kind of prior information about the data is needed. He then argued that it is necessary to use a mechanistic model that fits the data better than piecewise linear models in order to classify long-term dynamical properties from nonlinear finite time-series data. He also suggested that such a procedure seems necessary but is not sufficient.

The authors of these articles are former students, collaborators, conference co-organizers, and co-authors of Mimmo Iannelli, and the breadth of their contributed papers to this special issue will be a long-lasting testament to the breadth of Mimmo's own work and legacy.

The following rhyme was shown during the closing of the conference as a tribute to Mimmo from all the participants.

## Mimmorabilia

To Trento many of us came;

others couldn't … what a shame!

Great new problems to frame,

was our declared game.

But Mimmo's birthday celebration

was the *real* motivation;

thus to this destination

did we come from many-a-nation:

France and Finland and Bulgaria,

UK, Spain, Italia,

USA, Sweden and China,

Germany, Korea and Argentina,

Hungary, Netherlands, Algeria,

and last-but-not-least Romania.

Friendship, science, great libation,

filled this worthy celebration.

Now it's over, with consternation,

– back to my adoptive nation.

But we do not leave sad at all,

as in this meeting we had a ball!

Next occasion that is this tall

rest assured that we will call.

So, dear Mimmo, I say good-bye,

and you can tell this is no lie:

The situation is cut-and-dry,

I'll be your friend until I die.

(F.A. Milner, December 2011)

We are very grateful to Jim Cushing for making the arrangements for the publication of this collection of works, and we look forward to more such collections of works on innovative methods and models.

